# Gastrodin Improves the Activity of the Ubiquitin–Proteasome System and the Autophagy–Lysosome Pathway to Degrade Mutant Huntingtin

**DOI:** 10.3390/ijms25147709

**Published:** 2024-07-14

**Authors:** He Sun, Miao Li, Yunling Li, Na Zheng, Jiaxin Li, Xiang Li, Yingying Liu, Qianyun Ji, Liping Zhou, Jingwen Su, Wanxu Huang, Zhongbo Liu, Peng Liu, Libo Zou

**Affiliations:** 1 Department of Pharmacology, Shenyang Pharmaceutical University, 103 Wenhua Road, Shenhe District, Shenyang 110016, China; hesun1999@163.com (H.S.); 19863348219@163.com (M.L.); 15552371335@163.com (N.Z.); liuying568@yeah.net (Y.L.); jqy17749855497@163.com (Q.J.); zlp13050245797@163.com (L.Z.); 2 Wuya College of Innovation, Shenyang Pharmaceutical University, 103 Wenhua Road, Shenhe District, Shenyang 110016, China; ll1946842634@163.com (Y.L.); huangwx@syphu.edu.cn (W.H.); 3 Department of Bioengineering, Shenyang Pharmaceutical University, 103 Wenhua Road, Shenhe District, Shenyang 110016, China; 13840646328@163.com; 4 Department of Medicinal Chemistry, Shenyang Pharmaceutical University, 103 Wenhua Road, Shenhe District, Shenyang 110016, China; 13390178665@163.com; 5 School of Traditional Chinese Materia Medica, Shenyang Pharmaceutical University, 103 Wenhua Road, Shenhe District, Shenyang 110016, China; yykfire2024@163.com; 6 School of Pharmacy, Shenyang Pharmaceutical University, 103 Wenhua Road, Shenhe District, Shenyang 110016, China; liuzhongbo@syphu.edu.cn

**Keywords:** gastrodin, mutant huntingtin protein, ubiquitin–proteasome system, autophagy–lysosome pathway, neurodegeneration

## Abstract

Gastrodin (GAS) is the main chemical component of the traditional Chinese herb *Gastrodia elata* (called “Tianma” in Chinese), which has been used to treat neurological conditions, including headaches, epilepsy, stroke, and memory loss. To our knowledge, it is unclear whether GAS has a therapeutic effect on Huntington’s disease (HD). In the present study, we evaluated the effect of GAS on the degradation of mutant huntingtin protein (mHtt) by using PC12 cells transfected with N-terminal mHtt Q74. We found that 0.1–100 μM GAS had no effect on the survival rate of Q23 and Q74 PC12 cells after 24–48 h of incubation. The ubiquitin–proteasome system (UPS) is the main system that clears misfolded proteins in eukaryotic cells. Mutated Htt significantly upregulated total ubiquitinated protein (Ub) expression, decreased chymotrypsin-like, trypsin-like and caspase-like peptidase activity, and reduced the colocalization of the 20S proteasome with mHtt. GAS (25 μM) attenuated all of the abovementioned pathological changes, and the regulatory effect of GAS on mHtt was found to be abolished by MG132, a proteasome inhibitor. The autophagy–lysosome pathway (ALP) is another system for misfolded protein degradation. Although GAS downregulated the expression of autophagy markers (LC3II and P62), it increased the colocalization of LC3II with lysosomal associated membrane protein 1 (LAMP1), which indicates that ALP was activated. Moreover, GAS prevented mHtt-induced neuronal damage in PC12 cells. GAS has a selective effect on mHtt in Q74 PC12 cells and has no effect on Q23 and proteins encoded by other genes containing long CAGs, such as Rbm33 (10 CAG repeats) and Hcn1 (>30 CAG repeats). Furthermore, oral administration of 100 mg/kg GAS increased grip strength and attenuated mHtt aggregates in B6-hHTT130-N transgenic mice. This is a high dose (100 mg/kg GAS) when compared with experiments on HD mice with other small molecules. We will design more doses to evaluate the dose–response relationship of the inhibition effect of GAS on mHtt in our next study. In summary, GAS can promote the degradation of mHtt by activating the UPS and ALP, making it a potential therapeutic agent for HD.

## 1. Introduction

Huntington’s disease (HD) is an autosomal dominant neurodegenerative disease characterized by abnormal behavior, such as chorea, dystonia, cognitive impairment, and even psychiatric disturbances [1,2]. It is caused by an abnormal expansion of the CAG trinucleotide repeat in exon 1 of the Huntingtin gene (*IT15*) on chromosome 4p16.3 [3]. Thirty-six to thirty-nine CAG repeats will cause HD to manifest at reduced penetrance, and individuals may or may not develop HD symptoms. When the CAG repeat number is more than forty (mutant huntingtin, mHtt), HD occurs at full penetrance and patients develop HD symptoms [4].

Intracellular mutated and misfolded protein degradation occurs mainly through two systems, the ubiquitin–proteasome system (UPS) and the autophagy–lysosome pathway (ALP) [5]. The UPS is a mechanism that regulates or labels abnormal or excess proteins within cells. Polyubiquitinated proteins undergo modifications and are degraded in proteasomes. The UPS is composed of Ub (ubiquitin), E1 (ubiquitin activating enzyme), E2 (ubiquitin binding enzyme), E3 (ubiquitin protein ligase), and 26S proteasomes. The process of protein degradation mediated by the UPS includes the following: E1 binds to Ub to form the Ub–E1 complex, the Ub–E1 complex interacts with E2, E2 replaces El to form the Ub–E2 complex, and the Ub–E2 complex interacts with E3 to connect ubiquitin to the target protein for degradation [6]. Ubiquitinated misfolded proteins are recognized by the proteasome complex and then degraded [7]. In ALP, P62 binds LC3 to ubiquitinated substrates that are then degraded in autophagosomes. Autophagosomes fuse with lysosomes, and then the proteins or organelles are degraded by lysosomal enzymes [8].

UPS dysfunction can be directly induced by mHtt [9,10], which disrupts vesicular transport, blocks autophagosome–lysosome fusion, downregulates neuronal autophagy levels, and finally leads to neuronal death [11]. It has been reported that, compared with the ALP, the UPS may play a more important role in clearing N-terminal-truncated Huntington protein [12].

*Gastrodia elata* (called “Tianma” in Chinese) is a famous herb and medicine that has been used in China for centuries to treat many neurological conditions, including headaches, dizziness, spasms, epilepsy, stroke, and memory loss [13]. More than 80 kinds of phenols, polysaccharides, organic acids, and sterols have been isolated from *Gastrodia elata* [14]. Gastrodin (GAS), a phenolic glycoside, is considered the main bioactive component of *Gastrodia elata* [15]. GAS has many pharmacological effects, including sedation-hypnosis [16], analgesia [17], antiepilepsy [18], antidepressant [19], memory improvement [20], and antiaging [21,22]. The prominent effect of GAS on the central nervous system (CNS) suggests that it may be a potentially valuable therapeutic agent for neurodegenerative disease treatment.

*Gastrodia elata* can decrease mHtt-induced protein aggregations and attenuated mitochondrial dysfunction in 109Q transfected cells [23,24]. A phenolic compound of *Gastrodia elata* called para-hydroxybenzyl alcohol can effectively delay the progression of HD in *C. elegans* [25]. However, to our knowledge, it is unclear whether GAS exerts a therapeutic effect on HD. In the present study, we evaluated the effect of GAS on mHtt degradation and the UPS and ALP, which are the main systems for misfolded protein degradation, by using PC12 cells transfected with N-terminal mHtt Q74. We also evaluated the effect of GAS on grip strength and mHtt aggregates in HD transgenic mice (B6-hHTT130-N mice).

## 2. Results

### 2.1. Gastrodin Prevents mHtt Toxicity and Morphological Damage in Q74 PC12 Cells

**Figure 1:**
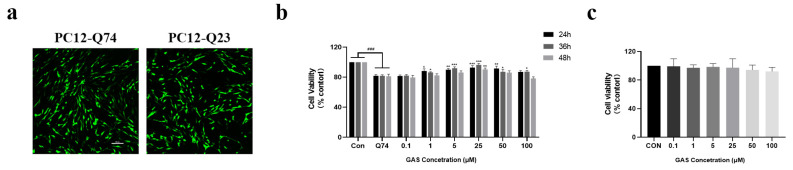
Gastrodin prevents mHtt toxicity in Q74 PC12 cells. (**a**) Schematic diagram of fluorescence transfection efficiency. (**b**) GAS enhanced the survival rate of Q74 PC12 cells. (**c**) No toxicity was observed in PC12 cells treated with 0.1–100 μM GAS for 36 h. All results are expressed as the mean ± SEM. *n* = 3. Scale Bar = 100 μm. ### *p* < 0.001 versus the control group. * *p* < 0.05, ** *p* < 0.01, *** *p* < 0.001 versus the Q74 group.

First, PC12 cells were transfected with GFP-labeled Htt with Q23 or Q74 repeats of the HTT partial exon 1, and the transfected cells showed bright green signals, indicating the formation of Htt as well as mHtt in the nucleus and cytoplasm (Figure 1a). The transfection efficiency was approximately 80–90%. Then, a CCK-8 assay was used to test cell viability. Compared with PC12 cells, cells transfected with Q74 showed obvious toxicity. When the GAS dose was higher than 1 μM, GAS modestly enhanced survival rate, but with statistical significance (*p* < 0.05, Figure 1b). Compared with the control group, PC12 cells transfected with Q23 did not show obvious toxicity (Supplementary Figure S1). No toxicity was observed in PC12 cells treated with 0.1–100 μM GAS for 36 h (Figure 1c).

**Figure 2:**
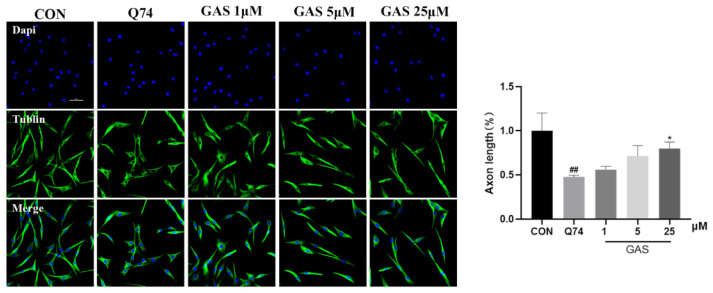
Gastrodin promotes neurite outgrowth in Q74 PC12 cells. All results are expressed as the mean ± SEM. *n* = 3. Scale Bar = 50 μm. ## *p* < 0.01 versus the control group; * *p* < 0.05 versus the Q74 group.

In order to observe the morphological effects of GAS on the morphology of Q74 PC12 cells, the cells were treated with different concentrations of GAS (1, 5, and 15 μM) for 36 h after the cells were grown to about 60%, and then immunofluorescence staining was performed using β-tubulin antibody, followed by observation of the cell morphology. We found that PC12 cells transfected with Q74 showed very short neurites. GAS tends to promote neurite length in Q74 PC12 cells (Figure 2).

### 2.2. Gastrodin Promotes mHtt Degradation in Q74 PC12 Cells

**Figure 3:**
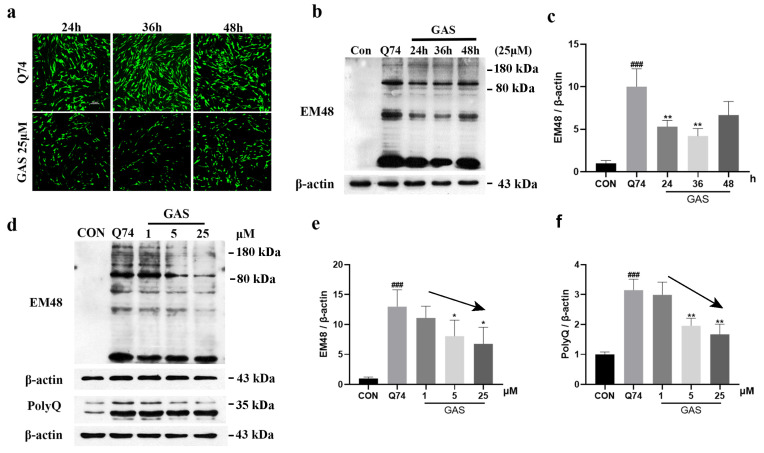
Gastrodin regulates mHtt level in Q74 PC12 cells. (**a**) Representative image of pEGFP-Q74 fluorescence at 24, 36, and 48 h after 25 μM GAS treatment. (**b**,**c**) GAS treatment for 24, 36, and 48 h downregulated the expression of mHTT (EM48). (**d**–**f**) Treatment of Q74 PC12 cells with different concentrations of GAS (1, 5, 25 μM) for 36 h downregulated the expression of mHTT (EM48) and polyQ. The black arrow indicates a dose-response curve to provide a clearer dose-dependent effect of GAS. All results are expressed as the mean ± SEM. *n* = 4. Scale Bar = 100 μm. ### *p* < 0.001 versus the control group. * *p* < 0.05, ** *p* < 0.01 versus the Q74 group.

Abnormally amplified polyQ sequences produce mHtt, further inducing cell toxicity. Therefore, we investigated the effect of GAS on the expression levels of mHtt and polyQ proteins in Q74 PC12 cells in order to evaluate whether GAS increased the survival rate of Q74 PC12 cells by promoting mHtt degradation. Q74 PC12 cells were incubated with 25 μM GAS for 24 to 48 h, the total intracellular mHtt protein levels were significantly decreased at 24 and 36 h (*p* < 0.01), and there was a decreasing trend at 48 h, though with no statistical difference (Figure 3a–c). Subsequently, the expression levels of mHtt and polyQ proteins were tested in Q74 PC12 cells treated with 1, 5 and 25 μM GAS for 36 h. The Western blotting results showed that GAS significantly reduced mHtt levels as detected with anti-mHtt or anti-polyQ antibodies in a dose-dependent manner (*p* < 0.05, Figure 3d–f).

### 2.3. Gastrodin Increases UPS Activity in Q74 PC12 Cells

**Figure 4:**
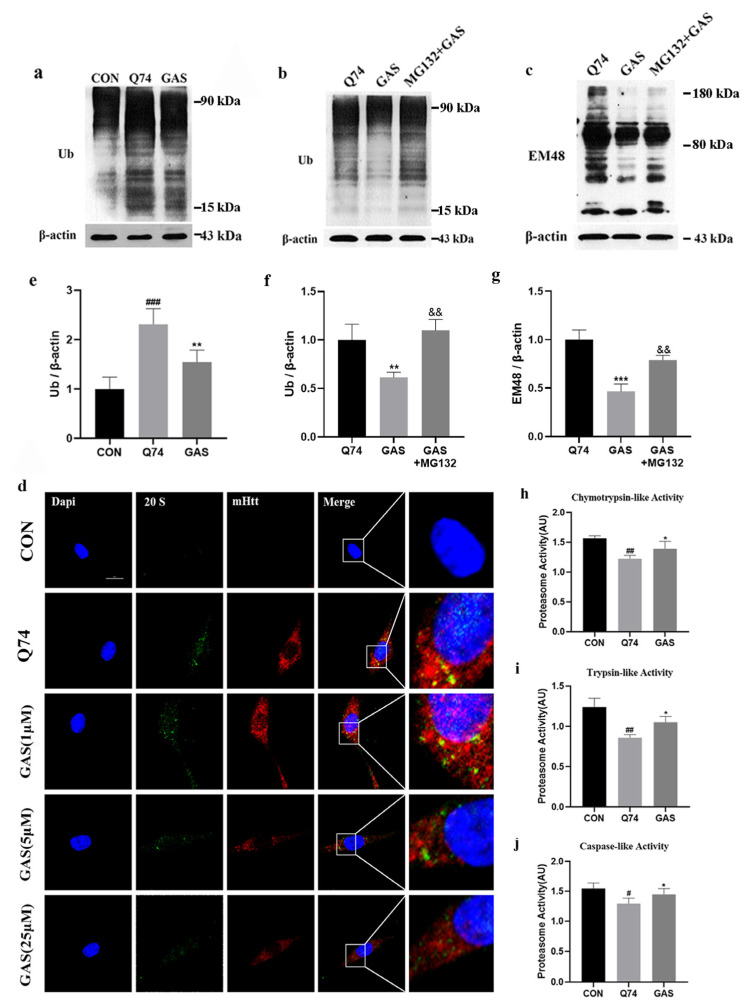
Gastrodin increases UPS activity in Q74 PC12 cells. (**a**,**e**) Treatment with 25 μM GAS for 36 h downregulated the expression of Ub in Q74 PC12 cells. (**b**,**c**,**f**,**g**) GAS downregulated the expression of Ub and mHtt in Q74 PC12 cells, which was blocked by MG132. (**d**) Representative image of the colocalization of the 20S proteasome and mHtt in Q74 PC12 cells, which were incubated with 25 μM GAS for 36 h. (**h**–**j**) GAS decreased chymotrypsin-like, trypsin-like and caspase-like peptidase activity in Q74 PC12 cells. All results are expressed as the mean ± SEM. *n* = 4. Scale Bar = 20 μm. For (**e**,**h**,**i**,**j**): # *p* < 0.05, ## *p* < 0.01, ### *p* < 0.001 versus the control group; * *p* < 0.05, ** *p* < 0.01 versus the Q74 group. For (**f**,**g**): ** *p* < 0.01, *** *p* < 0.001 versus the Q74 group; && *p* < 0.01 versus the GAS group.

The UPS is a mechanism that regulates, labels and clears abnormal or excess proteins within cells. Therefore, we investigated whether the UPS participates in GAS-induced mHtt degradation. Compared with that in the control group, the total amount of ubiquitinated proteins (Ub) in Q74 PC12 cells was significantly increased. Treatment with 25 μM GAS for 36 h significantly decreased the total amount of Ub (*p* < 0.01, Figure 4a,e). MG132 is a proteasome inhibitor. We used MG132 to inhibit the UPS and evaluated whether MG132 can block the effect of GAS on mHtt degradation in Q74 PC12 cells. We added 10 μM MG-132 together with 25 μM GAS into the cells for 36 h. The results show that MG132 significantly increased the total amount of Ub and the expression level of mHtt, confirming that GAS degrades mHtt by activating the UPS (*p* < 0.01, Figure 4b,c,f,g).

The 20S proteasome, an essential component of the UPS, degrades some of the oxidized proteins and misfolded proteins in a ubiquitin-independent manner [21]. We had already found that GAS can activate the UPS, and so we investigated whether this effect is mediated by the removal of the core 20S subunit blockade caused by mHtt. The 20S proteasome was fused in PC12 cells. After transfection with the Q74 plasmid, the expression of 20S proteasome was significantly increased, and widely distributed with mHTT, which may lead to impaired UPS function. GAS treatment attenuated these abnormal distributions (Figure 4d). Thus, GAS can improve the aggregation of the 20S proteasome induced by mHtt.

The 20S proteolytic core particle exerts chymotrypsin-like, trypsin-like and caspase-like effects on ubiquitinylated proteins, so we further tested the activity of these three peptidases in GAS-treated Q74 PC12 cells. The fluorescent peptide substrates were incubated with the cell lysate for 1 h. Compared with Q74 PC12 cells, cells treated with 25 μM GAS treated for 36 h exhibited significantly increased chymotrypsin-like, trypsin-like and caspase-like peptidase activities (*p* < 0.05, Figure 4h–j).

### 2.4. Gastrodin Actives ALP in Q74 PC12 Cells

**Figure 5:**
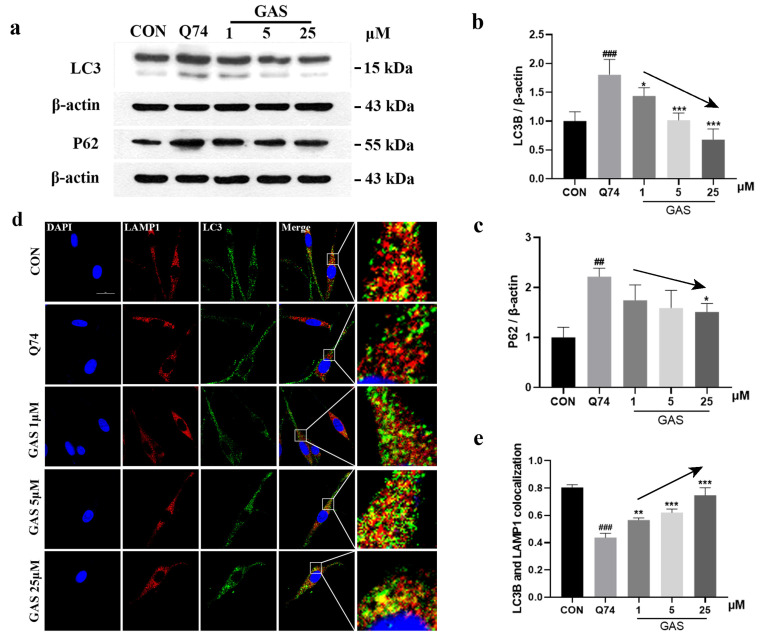
Gastrodin actives the ALP in Q74 PC12 cells. (**a**–**c**) GAS treatment for 36 h downregulated the expression of LC3-II and P62 in Q74 PC12 cells. (**d**) Representative image of the colocalization of LC3-II and LAMP1 in Q74 PC12 cells, which were incubated with 25 μM GAS for 36 h. (**e**) Quantitative analysis of the Mander’s overlap coefficient of LC3-II and LAMP1 in Q74 PC12 cells. The black arrow indicates a dose-response curve to provide a clearer dose-dependent effect of GAS. All results are expressed as the mean ± SEM. *n* = 3. Scale Bar = 20 μm. ## *p* < 0.01, ### *p* < 0.001 versus the control group. * *p* < 0.05, ** *p* < 0.01, *** *p* < 0.001 versus the Q74 group.

The ALP is another system for misfolded protein degradation. WB results show that GAS treatment for 36 h significantly downregulated the expression of LC3-II and P62 in Q74 PC12 cells in a dose-dependent manner (*p* < 0.05, Figure 5a–c). It is generally believed that the increase in LC3-II levels and the decrease in P62 levels indicate an increase in intracellular autophagy flux. However, the relationship between LC3-II and SQSTM1 P62 levels is not fixed [26]. To continue exploring whether GAS reduced mHtt through the ALP, we determined the colocalization levels of LC3-II and a lysosomal membrane protein, LAMP1. The colocalization of LAMP1 and LC3-II is a marker for ALP activation [27,28]. The immunofluorescence results show that treatment with GAS results in more colocalization between LAMP1 and LC3-II in Q74 PC12 cells, which indicates the formation of autophagic lysosomes; thus, the ALP was activated (Figure 5d,e).

### 2.5. Gastrodin Does Not Alter the Levels of Normal Q23 HTT and Other CAG Repeat Proteins

**Figure 6:**
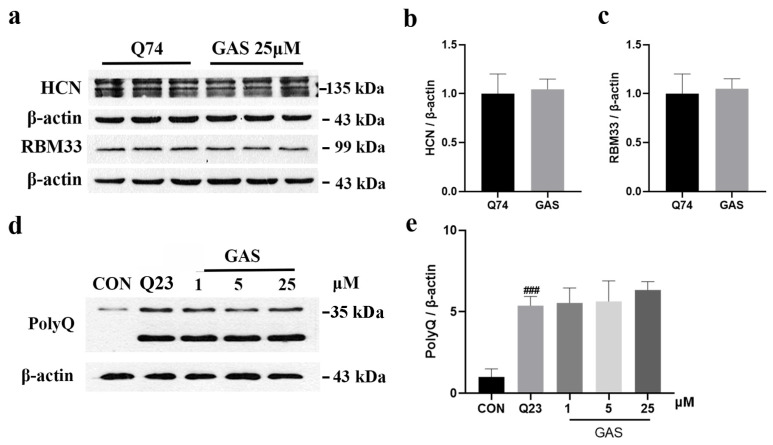
Gastrodin does not alter the normal Huntington protein or other types of CAG repeat proteins. (**a**–**c**) There were no differences in the expression of HCN1 and RBM33 between Q74- and GAS-treated cells. (**d**,**e**) GAS treatment for 24 h did not alter the expression of polyQ in Q23 PC12 cells. All results are expressed as the mean ± SEM. *n* = 3. ### *p* < 0.001 versus the control group.

Previous studies have shown that CAG repeat sequences are also present in RBM33 (10 CAG repeats) and HCN1 (>30 CAG repeats) [29,30]. Therefore, we treated PC12 cells with 25 μM GAS for 36 h to detect whether GAS specifically induces mHtt degradation. WB results show that there was no significant difference in the expression of RBM33 or HCN proteins between Q74-transfected PC12 cells and GAS-treated Q74 PC12 cells (Figure 6a–c). Subsequently, we evaluated whether GAS can decrease normal Htt levels in cells. The results show that there was no significant difference in the expression of polyQ between Q23-transfected PC12 cells and GAS-treated Q23 PC12 cells (Figure 6d,e). These results indicate that GAS specifically induced mHtt degradation and did not alter normal Htt or other CAG repeat proteins.

### 2.6. Gastrodin Increases Grip Strength and Decreases mHTT Aggregates in B6-hHTT130-N Mice

**Figure 7:**
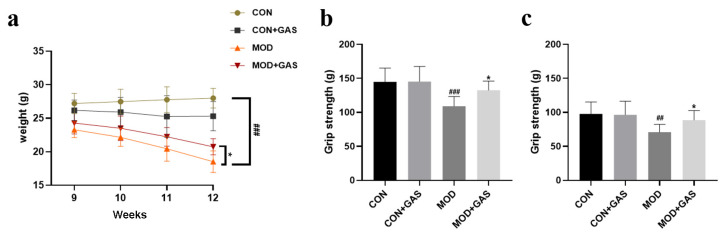
Gastrodin attenuates body weight loss and muscle strength weakness in B6-hHTT130-N mice. (**a**) B6-hHTT130-N mice exhibited a progressive decrease in body weight, and treatment with 100 mg/kg GAS attenuated body weight loss. B6-hHTT130-N mice exhibited a decrease in front paw grip strength (**b**) and four paw grip strength (**c**), and treatment with 100 mg/kg GAS improved muscle strength. The control group (CON) was C57BL/6 mice, and the model group (MOD) was B6-hHTT130-N mice. All results are expressed as the mean ± SEM. *n* = 10. ## *p* < 0.05, ### *p* < 0.001 versus the control group. * *p* < 0.05 versus the model group.

The body weight of mice can reflect their general health. We found that the body weight of B6-hHTT130-N mice progressively decreased from 23.28 ± 1.05 g to 18.51 ± 0.92 g [Fweek = 10.571, *p* < 0.001; Fgroup = 105.548, *p* < 0.001; Fweek × group = 3.713, *p* < 0.001, Figure 7a]. Treatment with 100 mg/kg GAS significantly attenuated the body weight loss of B6-hHTT130-N mice (*p* < 0.01, Figure 7a). B6-hHTT130-N mice contain 130 CAG repeats in the *IT15* gene, showing an mHtt aggregation from 10 weeks of age and movement disorders from 12 weeks of age. Both the front paw grip strength and four paw grip strength were decreased in 12-week-old B6-hHTT130-N mice compared with WT mice [front paw grip strength: Fgenotype = 11.728, *p* < 0.001; Ftreatment = 2.605, *p* = 0.115; Fgenotype × treatment = 3.496, *p* = 0.070, Figure 7b] [four paw grip strength: Fgenotype = 18.397, *p* < 0.001; Ftreatment = 4.431, *p* < 0.05; Fgenotype × treatment = 4.068, *p* = 0.051, Figure 7c]. Treatment with 100 mg/kg GAS significantly attenuated muscle strength weakness (*p* < 0.05, Figure 7b,c).

**Figure 8:**
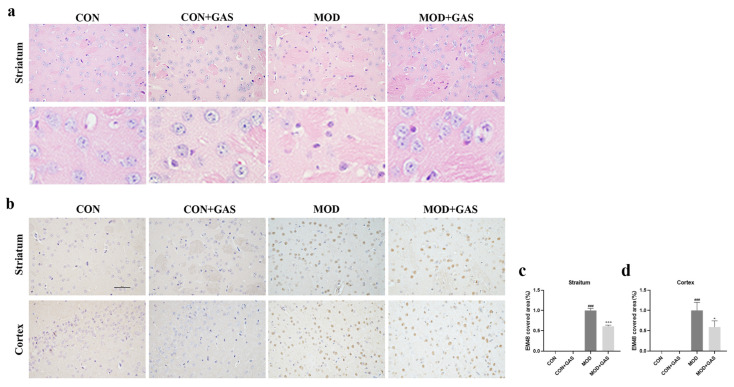
Gastrodin attenuates neuronal injury and decreases mHtt aggregates in B6-hHTT130-N mice. (**a**) HE-stained sections showing pathological changes in the striatum and cerebral cortex of mice in each group. (**b**–**d**) Treatment with 100 mg/kg GAS significantly decreased the positive area of EM48-positive cells in the striatum and cerebral cortex of B6-hHTT130-N mice. The control group (CON) was C57BL/6 mice, and the model group (MOD) was B6-hHTT130-N mice. All results are expressed as the mean ± SEM. *n* = 5. Scale bar = 100  μm. ### *p* < 0.001 versus the control group. * *p* < 0.05, *** *p* < 0.001 versus the model group.

HE staining showed that there was complete cell structure and large and regular nuclei in the striatum of WT and WT + GAS mice. However, in the striatum of B6-hHTT130-N mice, there was extensive neuronal shrinkage and nuclear chromatin condensation. Treatment with 100 mg/kg GAS could attenuate the neuronal morphology (Figure 8a). Immunohistochemistry results show that there was no positive staining in the striatum and cerebral cortex of WT and WT + GAS mice (Figure 8b–d). In the striatum and cerebral cortex of B6-hHTT130-N mice, the mHtt-positive stained area was significantly increased (*p* < 0.001, Figure 8b–d). GAS significantly decreased the mHtt-positive staining area (*p* < 0.05, Figure 8b–d). This is a high dose (100 mg/kg GAS) compared with experiments on HD mice with other small molecules. We will design more doses to evaluate the dose–response relationship of inhibition effect of GAS on mHtt in our next study.

## 3. Discussion

*Gastrodia elata* has been used as an herbal medicine for a thousand years in China. In Chinese pharmacopeia, both GAS and p-hydroxybenzyl alcohol (HBA) are used as the phytochemical markers of G. elata to control the quality of *Gastrodia elata* [31], which indicates that GAS is the main component of *Gastrodia elata*. As the main chemical component of *Gastrodia elata*, GAS has been proven to effectively treat neurodegenerative diseases [21]. GAS alleviated memory deficits and decreased the levels of Aβ and p-Tau in the hippocampus of mice intracerebroventricularly injected with Aβ1-42 [32]. GAS attenuated spatial memory deficits and decreased Aβ deposition in the brains of transgenic Tg2576 mice, which are used to mimic Alzheimer’s disease [33]. GAS could prolong lifespan and rescue climbing ability and dopaminergic neuron numbers in the brains of Pink1B9 mutant flies, a Parkinson’s disease model [34]. GAS suppressed the NLRP3 inflammasome, reduced pyroptosis, and exerted neuroprotective effects in traumatic brain injury rats [35]. GAS can restore cognitive behaviors and promote neurogenesis in the hippocampus in bilateral common carotid artery occlusion mice [36]. *Gastrodia elata* can decrease Htt-induced protein aggregations and has been shown to attenuate mitochondrial dysfunction in 109Q transfected cells [23,24]. Para-hydroxybenzyl alcohol, a phenolic compound extracted from *Gastrodia elata*, can effectively delay the progression of HD in *C. elegans* [25]. HD is the most common polyglutamine disease, and abnormal mHtt aggregates are the main pathogenesis. Inhibiting and clearing mHtt aggregates are important methods to attenuate HD-like symptoms. To our knowledge, it is unclear whether GAS exerts a therapeutic effect on HD. This is the first in vivo study to evaluate the effect of the *Gastrodia elata* component on HD. HD is caused by a CAG repeat expansion in the Htt gene, and results in the expression of mHtt. Therefore, to study the pharmacological effect of compounds in HD, the in vitro models should express mHtt. We believe HD patient iPSC-derived neurons and primary neurons from HD transgenic mice are the best in vitro models, while cell lines transfected with plasmids containing a pathological CAG-repeat of the HTT exon 1 are acceptable (convenient acquisition, not involving ethics) in vitro models.

The accumulation of misfolded and dysfunctional proteins is one of the causes of neurodegenerative diseases. In the present study, we found that GAS can significantly promote the degradation of the mHtt protein in Q74-transfected PC12 cells and in the striatum and cerebral cortex of B6-hHTT130-N mice. The UPS is the most famous protein degradation pathway and is capable of identifying misfolded proteins and further degrading these abnormal proteins. In the UPS, ubiquitin is a signaling molecule that marks target proteins, the proteasome is the site for target protein degradation, E3 ligases have special recognition of target proteins, and various deubiquitination enzymes promote ubiquitin recycling and maintain the normal operation of the UPS. UPS dysfunction affects the clearance of unfolded and misfolded toxin proteins. The literature reports that, in regulating mHtt degradation, the UPS plays a more important role than the ALP [37]. The UPS is dysfunctional in HD [38], and mHtt decreases the proteasomal activity of the UPS in brain cells and skin fibroblasts, which is accompanied by mitochondrial membrane potential loss and the colocalization of the 20S proteasome with mHtt aggregates [19,39,40]. Overexpression of the proteasome activator can increase neuron viability in HD [41]. In this study, GAS significantly reversed the loss of UPS activity induced by mHtt and reduced the colocalization of mHtt and the 20S proteasome in Q74 PC12 cells. MG132, a ubiquitin proteasome inhibitor, can increase the total amount of Ub and inhibit the effect of GAS when downregulating the expression of mHtt. We also found that GAS increased the activities of three peptidases, including chymotrypsin-like, caspase-like, and trypsin-like peptidases in Q74 transfected PC12 cells. These results confirm that GAS degraded mHtt by activating the UPS. GAS did not alter normal Htt or other CAG repeat proteins, which indicates that GAS had the specific function of inducing mHtt degradation and had no effect on normal healthy neuronal cells. In vivo, we found that treatment with 100 mg/kg GAS prevented weight loss and significantly attenuated the muscle weakness of the front paws and four paws in B6-hHTT130-N mice. GAS also decreased mHtt aggregates in the striatum and cerebral cortex of B6-hHTT130-N mice, which indicates that GAS still maintains suitable anti-HD effects in vivo.

Misfolded proteins are engulfed by autophagosomes and then transported to lysosomes for degradation [42]. In addition to the UPS, the ALP is another system that mediates intracellular mutated and misfolded protein degradation in eukaryotic cells. Studies have shown that impairing autophagic function causes striatal medium spiny neuron degeneration in HD patients [43]. Restoration of autophagy flux can decrease mHtt levels and toxicity [44]. Autophagy dysfunction in HD patients leads to impaired synaptic maintenance and thus early manifestations of disease [45]. Upregulation of let7b miRNA increases LAMP2A levels and reduces extended polyQ levels in mouse striatal cells [46]. In this study, Q74-transfected PC12 cells treated with GAS showed abnormal autophagic flux, and the expression of LC3-II and P62 was decreased in a dose-dependent manner. However, GAS significantly increased the colocalization of LC3-II and LAMP1; thus, GAS promotes the fusion of autophagosomes with lysosomes. It has been shown that mHtt co-aggregates with transcription factor EB (TFEB), a major regulator of lysosomal biogenesis and autophagy, and possibly interferes with its function. GAS could activate TFEB in foam cells [47]. Consequently, it is possible that TFEB acts as a target for GAS, an idea which requires further experimentation in to confirm. Next, we will evaluate whether GAS can improve the activity of the UPS and ALP in B6-hHTT130-N mice. In addition, axons are the structural basis for synaptic transmission, transmitting nerve impulses from the cell body to other neurons. There are substantial synaptic and axonal function impairments in HD [48]. Our experimental results show that GAS attenuated mHtt-induced cell morphology damage. GAS also attenuated mHtt-induced neuronal loss in B6-hHTT130-N mice, and its neuroprotective effect will also be further explored. For an in vivo study, 100 mg/kg GAS is a high dose when compared with experiments on HD mice with other small molecules. However, 100 mg/kg is still an acceptable dose during the pharmacodynamic evaluation process [49,50,51]. We intend to design more doses in order to evaluate the dose–response relationship of the inhibition effect of GAS on mHtt in our next study. Wang Q et al. have reported that GAS can quickly cross the BBB, but that the ratios of AUC (brain)/AUC (plasma) are not high and that the levels of GAS decline rapidly after intravenous injection [52]. Pharmacokinetics may limit the efficacy and clinical development prospects of GAS. Therefore, sustained-release drug delivery or site-specific brain drug delivery may have more advantages for GAS.

There were some limitations to this study. Rat adrenal pheochromocytoma cell-derived PC12 cell line has some characteristics of neuronal cells and is widely used in cell biology. However, PC12 cell is not a real neuronal cell and has significant differences from primary neuronal cells [53]. The caudate nucleus and putamen are significantly affected in primate brains with HD but are indistinguishable in rodents [54]. GAS has adverse reactions related to the digestive system and to skin allergies and also has a sedative effect, which may be induced by the off-target effects of GAS. However, in this study, we did not observe these adverse reactions. The absence of a positive control is a defect of this study. Effective therapeutic drugs for HD are rare, prompting us to consider whether some authorized natural compounds (curcumin, resveratrol), which have been reported to decrease the expression of mHtt, should be used as positive controls.

**Figure 9:**
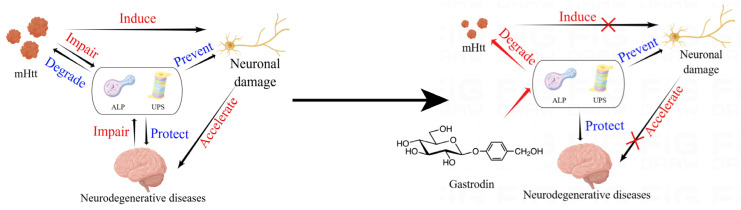
Gastrodin improves the activity of the UPS and ALP in 74Q mHtt cells and attenuates muscle strength weakness in B6-hHTT130-N mice (By Figdraw).

In summary, we have demonstrated, for the first time, that GAS can specifically clear mHtt in human mHtt-transfected PC12 cells and in the striatum and cerebral cortex of B6-hHTT130-N mice, an HD model transgenic mouse, by activating the UPS and ALP and reducing the toxicity of mHtt (Figure 9), making it a potential therapeutic agent for HD.

## 4. Materials and Methods

### 4.1. Materials

Gastrodin (G299059, purity >98%) was purchased from Aladdin (Shanghai, China). pEGFP-Q23 and pEGFP-Q74 were gifts from David Rubinsztein (Addgene plasmid # 40261; http://n2t.net/addgene:40261, Avalilable at: URL Accessed 20 May 2024; RRID: Addgene_40261. Addgene plasmid # 40262; http://n2t.net/addgene:40262, Avalilable at: URL Accessed 20 May 2024; RRID: Addgene_40262) [55]. RPMI-1640 medium was purchased from Servicebio (Wuhan, China). MG132 (M126521) and Suc-Leu-Leu-Val-Tyr-AMC (S380686) were purchased from Aladdin (Shanghai, China). Proteasome substrate II (539141) and tryptase substrate (SCP0257) were purchased from Sigma Aldrich (St. Louis, MO, USA). The antibodies are listed in Table 1.

### 4.2. Animals

Male 7-week-old B6/JGpt-Tg(hHTT-CAG130)90/Gpt mice (Strain NO.T054804) were purchased from GemPharmatech (Nanjing, China), and wild-type mice were used as controls. Mice were housed under a 12 h light/dark cycle and had free access to food and water. All animal studies were performed in strict accordance with Chinese legislation on the use and care of laboratory animals and the guidelines established by the Institute for Experimental Animals at Shenyang Pharmaceutical University.

### 4.3. Drug and Treatment Schedule

The mice were divided into four groups: WT group, WT and 100 mg/kg gastrodin dose group, B6-hHTT130-N group, and B6-hHTT130-N and 100 mg/kg gastrodin dose group. Each group had 10 mice. Gastrodin was dissolved in 0.5% CMC-Na. Eight-week-old mice were given gastrodin by gavage once a day until 12 weeks of age. The dose of gastrodin was based on previous studies [36,56,57]. After the grip strength test, the mice were sacrificed by CO_2_ inhalation. The brains from five mice in each group were collected and paraffin embedded for HE staining and immunohistochemistry tests. The other brains were collected and stored at −80 °C.

### 4.4. Grip Strength Test

The mice were tested at 12 weeks of age. The back neck skin of the mouse was pinched and its front paws or four paws grabbed the dynamometer (Shanghai XinRuan Information Technology Co., Ltd., Shanghai, China). The mouse was slowly pulled back until it lost its grip and the reading of the dynamometer instrument was recorded. The average of the results of three consecutive tests was used for comparative analyses.

### 4.5. Cell Cultures and Viability

The PC12 pheochromocytoma cell line was obtained from the Cell Resource Center, Peking Union Medical College. Cells were cultured in RPMI 1640 medium supplemented with 10% fetal bovine serum at 37 °C and 5% CO_2_/95% air. PC12 cells were seeded into 96-well plates, cultured at 37 ℃ with 5% CO_2_, and transfected with 1 μg of GFP-tagged plasmid DNA containing a normal (Q23) or pathological CAG-repeat length (Q74) of the HTT partial exon 1 [55] using Lipofectamine 3000 (Invitrogen, CA, USA) according to the manufacturer’s protocol. Cell confluence was approximately 60–80% before infection, and the medium was replaced with basic medium that did not contain serum or antibiotics. Gastrodin was dissolved in serum free cell culture medium. After 24 h of cell transfection, different concentrations of GAS were added to the cells for 12 to 48 h. Then, 10 μL (10 nmol/L) CCK8 reagent (APEXBIO, Houston, USA) was added to the wells, the cells were cultured at 37 °C in 5% CO_2_ for 1 h, and the absorbance was measured at 460 nm (Thermo Fisher Scientific, Cleveland, OH, USA). Neurite length was defined as the distance from the center of the cell to the end of the neurite and was measured using ImageJ v1.8.0 in 5 random fields from each sample. The total length of neurites (the sum of all neurites’ length of a cell) and their means were recorded and normalized against those of the control group.

### 4.6. Western Blot Analysis

Different groups of cells were harvested and lysed with tissue lysis buffer [20 mM Tris-HCl buffer (pH 7.6), 150 mM NaCl, 2 mM EDTA·2Na, 50 mM sodium fluoride, 1 mM sodium vanadate, 1% NP-40, 1% sodium deoxycholate, 0.1% sodium dodecyl sulfate, 1 mg/mL pepstatin, 1 mg/mL aprotinin, and 1 mg/mL leupeptin]. The samples were centrifuged at 12,000 rpm at 4 °C for 20 min, and the supernatant was collected. A BCA protein detection kit was used to determine the protein concentration. Loading buffer was added to the supernatant and stored at −80 °C until use. Thirty micrograms of protein from cell lysates were run on a 10% sodium dodecyl sulfate polyacrylamide gel and electrophoretically transferred to a PVDF membrane (Millipore, Billerica, MA, USA). The PVDF membrane was incubated with the primary antibodies against EM48 (1:500), polyglutamine, ubiquitin, LC3B, P62, β-actin, RBM33 and HCN overnight at 4 °C and then incubated with the secondary antibodies at 37 °C for 1 h. Blots were visualized using an ECL kit (Beyotime Biotechnology, Shanghai, China). ImageJ software was used to determine the band density.

### 4.7. Immunofluorescence

The cells were fixed with 4% paraformaldehyde for 20 min, permeated with 0.1% Triton X-100 for 5 min, and blocked with 1% goat serum for 1 h. The cells were incubated with primary antibodies against 20S proteasome and EM48 or Lamp1 and LC3B or β-tublin at 4 °C overnight and then incubated with secondary antibodies (Table 2) at 37 °C for 1 h and DAPI (1:10,000, 4083S, Cell Signaling Technology, Danvers, MA, USA) at 37 °C for 10 min. Finally, specimens were examined under a confocal microscope (Nikon, Tokyo, Japan).

### 4.8. Proteasome Activity Assays

Different groups of cells were harvested and lysed in proteasome activity assay buffer. The samples were centrifuged at 12,000 rpm at 4 °C for 20 min, and the supernatant was collected. A BCA protein detection kit was used to determine the protein concentration. Twenty micrograms of protein from cell lysates were added to a 96-well plate. Then, the following fluorescent substrate was added to the well: Suc-Leu-Leu-Val-Tyr-AMC (40 µM) to test chymotrypsin-like activity, Z-Leu-Leu-Glu-AMC (40 µM) to test cysteine-like activity, and Ac-Arg-Leu-Arg-AMC (10 µM) to test trypsin-like activity. All three fluorescent substrates were dissolved in DMSO, then diluted in serum free cell culture medium. The final concentration of DMSO was <5%. After incubation in the dark at 37 °C for 1 h, the fluorescence was detected by a microplate reader (380 nm excitation, 460 nm emission).

### 4.9. Immunohistochemistry

Mouse brains were fixed with 4% paraformaldehyde, embedded in paraffin, and sliced into 5 µm sections. The sections were deparaffinized, treated with 3% H_2_O_2_ for 15 min and blocked in 10% normal goat serum for 1 h. The brain sections were incubated with the primary antibody against EM48 at 4 °C overnight and then washed with phosphate-buffered saline (PBS) three times. The sections were incubated with the biotin-labeled secondary antibodies at 37 °C for 1 h. Then, they were visualized using a DAB kit (Boster, Wuhan, China). The positive area of each section was quantified using ImageJ software.

### 4.10. HE Staining

Five-micron-thick brain sections were dewaxed in water and then placed in hematoxylin staining solution for 30 s. Staining was undertaken in acidic water for 10 s and then rinsed using running water for 5 min. After staining with eosin staining solution for 40 s, the brain sections were placed in 70% and 90% alcohol for 10 min each to dehydrate. Finally, the slices were sealed with neutral gum and dried before being photographed using an optical microscope.

### 4.11. Statistical Analysis

All analyses were performed using SPSS v23.0. Data are presented as the mean ± SEM. Differences between groups were analyzed using one-way or two-way ANOVA followed by Fisher’s LSD post hoc test in the case of homogeneous variance or Dunnett’s T3 test in the case of heterogeneous variance. A *p* value of < 0.05 was considered to indicate significance.

## Figures and Tables

**Table 1:** The antibodies used in this study.

Antibody	Source	Catalog No.
EM48	Sigma–Aldrich, Inc. St. Louis, MO. USA	MAB5374
Polyglutamine	Sigma–Aldrich, Inc. St. Louis, MO. USA	P1874
Ubiquitin	Santa Cruz Biotechnology, Santa Cruz, CA, USA	sc-166553
Lamp1	Santa Cruz Biotechnology, Santa Cruz, CA, USA	sc-20011
β-actin	Santa Cruz Biotechnology, Santa Cruz, CA, USA	sc-8432
LC3B	Zen-Bioscience, Chengdu, China	381,544
P62	Zen-Bioscience, Chengdu, China	380,612
RBM33	Bioss, Beijing, China	bs-21295R
HCN	Bioss, Beijing, China	bs-6604R
β-tubulin	Wanleibio, Shenyang, China	WL01931
Anti-rabbit IgG	Proteintech, Wuhan, China	20,000,730
Anti-mouse IgG	Abcam, Cambridge, UK	ab150107

**Table 2:** The antibodies’ dilution ratios.

Antibody	Dilution Ratio in WB	Dilution Ratio in IF or IHC
EM48	1: 500	1: 100
Polyglutamine	1: 500	
Ubiquitin	1:500	1:1000
Lamp1		1:100
β-actin	1:800	
LC3B	1:500	1:100
P62	1:500	
RBM33	1:500	
HCN	1:500	
20S proteasome		1:200
β-tubulin		1:500

## Data Availability

The data used to support the findings of this study are available from the corresponding authors upon request.

## Supplementary Materials

The following supporting information can be downloaded at: https://www.mdpi.com/article/10.3390/ijms25147709/s1.
