# Supplementary material for: Gastrodin Improves the Activity of the Ubiquitin–Proteasome System and the Autophagy–Lysosome Pathway to Degrade Mutant Huntingtin
Source: Int J Mol Sci. 2024 Jul 14;25(14):7709. doi: 10.3390/ijms25147709 (PMC11277377; doi:10.3390/ijms25147709)
Supplement: Supplementary file 1 [file ijms-25-07709-s001.zip › ijms-3069251-supplementary.pdf]

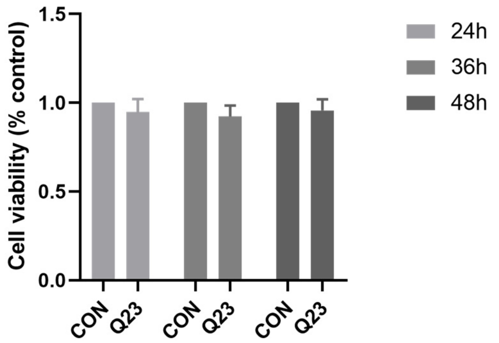

Supplementary Figure S1 PC12 cells transfected with Q23 didn't show obvious toxicity. All results are expressed as the mean ± SEM. n=3.
